# Debate: Why should gender-affirming health care be included in health science curricula?

**DOI:** 10.1186/s12909-020-1963-6

**Published:** 2020-02-14

**Authors:** Elma de Vries, Harsha Kathard, Alex Müller

**Affiliations:** 1 School of Public Health and Family Medicine, University of Cape Town, Cape Town, South Africa; 2 Department of Health and Rehabilitation Sciences, University of Cape Town, Cape Town, South Africa; 3 Gender, Health and Justice Research Unit, University of Cape Town, Cape Town, South Africa

**Keywords:** Transgender, Trans and gender diverse, Health disparities, Pathologisation, Gender-affirming health care, Social justice, Health science education

## Abstract

**Background:**

Every person who seeks health care should be affirmed, respected, understood, and not judged. However, trans and gender diverse people have experienced significant marginalization and discrimination in health care settings. Health professionals are generally not adequately prepared by current curricula to provide appropriate healthcare to trans and gender diverse people. This strongly implies that health care students would benefit from curricula which facilitate learning about gender-affirming health care.

**Main body:**

Trans and gender diverse people have been pathologized by the medical profession, through classifications of mental illness in the Diagnostic and Statistical Manual of Mental Disorders (DSM) and International Classification of Disease (ICD). Although this is changing in the new ICD-11, tension remains between depathologization discourses and access to gender-affirming health care.

Trans and gender diverse people experience significant health disparities and an increased burden of disease, specifically in the areas of mental health, Human Immunodeficiency Virus, violence and victimisation. Many of these health disparities originate from discrimination and systemic biases that decrease access to care, as well as from health professional ignorance.

This paper will outline gaps in health science curricula that have been described in different contexts, and specific educational interventions that have attempted to improve awareness, knowledge and skills related to gender-affirming health care. The education of primary care providers is critical, as in much of the world, specialist services for gender-affirming health care are not widely available. The ethics of the gatekeeping model, where service providers decide who can access care, will be discussed and contrasted with the informed-consent model that ﻿upholds autonomy by empowering patients to make their own health care decisions.

**Conclusion:**

There is an ethical imperative for health professionals to reduce health care disparities of trans and gender diverse people and practice within the health care values of social justice and cultural humility. As health science educators, we have an ethical duty to include gender-affirming health in health science curricula in order to prevent harm to the trans and gender diverse patients that our students will provide care for in the future.

## Background

Every person who seeks health care should be affirmed, respected, understood, and not judged. However, trans and gender diverse (TGD) people have experienced significant marginalization and discrimination in health care settings, as will be described further below. Health professionals are generally not adequately prepared by current curricula to provide healthcare to TGD people and have described feeling “completely out-at-sea” [1]. This strongly implies that healthcare students would benefit from curricula which facilitate learning about gender-affirming health care.

The literature search for this debate started with a key word search of databases including Scopus, Medline, Pubmed and Web of Science during the time period 2017–2018. Search terms included ‘trans’, ‘transgender’, ‘medical education’, ‘health science education’, ‘gender-affirming’, ‘curriculum’ and combinations thereof. A search of article reference lists identified further relevant articles as did personal communication with colleagues. This data informed the main topics for this debate.

Transgender is a term that refers to persons whose gender identity is different to that normatively expected on the basis of assigned sex. Gender diverse is a term to describe “people who do not conform to society’s or culture’s expectations for men and women” [2]. Nonbinary is a term used for a person who identifies as neither male nor female [3] and gender nonconforming for a person whose gender identity is different to that normatively expected on the basis of assigned sex, “but may be more complex, fluid, multifaceted, or otherwise less clearly defined than a transgender person” [3]. Genderqueer is another term used by some with this range of identities [3]. For this article, trans and gender diverse (TGD) will be used as an umbrella term to include transgender, gender nonconforming, genderqueer and gender diverse people. Cisgender is a term for someone whose gender identity is the same as that normatively expected on the basis of their assigned sex. Gender-affirming health care ﻿has been described by Radix, Reisner and Deutch [4] as “health care that holistically attends to transgender people’s physical, mental, and social health needs and well-being while respectfully affirming their gender identity”. This is more than just transition-related care and refers to an affirming experience in all health care encounters. Gender-affirming care models utilise an approach of depathologisation of human gender diversity (transgender as “identity”), rather than a pathological perspective (transgender as “disorder”) [4].

Until recently, little gender-affirming research existed, and, in the literature, TGD people have often been included in the broader grouping LGBT. This acronym combines sexual minority people (lesbian, gay, and bisexual people), and gender minority people (TGD people). These sexual and gender minority groups have in common that they often experience social exclusion, stigma, discrimination, violence, as well as ignorance from health professionals [5]. These experiences are rooted in societal heteronormativity and cisnormativity that generally marginalises non-heteronormative sexual (LGB) and gender (TGD) identities. Heteronormativity is “the assumption that everyone is heterosexual, and that heterosexuality is superior to all other sexualities” [6]. Cisnormativity is “the assumption all people are cisgender, that those assigned male at birth always grow up to be men and those assigned female at birth always grow up to be women” [7]. This strong normative facilitates transphobia, which is emotional disgust, fear, hostility, violence, anger or discomfort felt or expressed towards people who do not conform to the gender expectations of society [8]. Thus, transphobia has been described as a symptom of hetero-cis-normativity [9]. Müller comments that “though there is a common source of oppression [hetero-cis-normativity], it has to be acknowledged that this oppression acts on different identities (sexual orientation or gender) in different ways” [10].

Compared to cisgender people, TGD people experience significant health disparities and an increased burden of disease [11]. Many of these health disparities originate from discrimination and systemic biases that decrease access to care, as well as from health professionals’ ignorance [12]. It is thus critical to educate health professionals to deliver equitable care for TGD populations, but most health sciences education institutions do not yet provide sufficient education [13].

## Brief history of pathologisation, Diagnostic and Statistical Manual of Mental Disorders (DSM) and International Classification of Disease (ICD)

People with diverse gender identities and expressions have been part of society for millennia. With increasing medical interest in providing transition-related care in the 1950’s, the TGD person became a “patient” and with the “medical gaze”, diverse gender identities have often been viewed as pathology [14]. The history of pathologisation is important to understand in relation to gender-affirming health care, as there is a tension between pathologisation and access to health care [15].

Historically, medical research produced the “scientific” evidence that pathologized sexualities and gender identities that did not conform to societal expectations, as well as supported treatments such as so-called “conversion therapy” that is now regarded as unethical [15]. Until 1973, homosexuality was listed as a mental illness in the American Psychiatric Association’s Diagnostic and Statistical Manual of Mental Disorders (DSM) [16]. Sex between people of the same sex or gender still remains criminalised in 68 United Nations member states in 2019 [17]. The DSM is an influential document that is used internationally to diagnose and classify mental illness. Gender diversity remains listed in the DSM until today. In the DSM-4, the term “Gender identity disorder” was used and in DSM-5 this had been changed to “Gender Dysphoria” [18]. The intention of the change in the DSM-5 was to reduce stigma, while ensuring that individuals are able to access the care they need [14]. Proponents for the term “Gender Dysphoria” argued that it was less stigmatizing than “Gender identity disorder” [14]. However, others have pointed out that gender diversity in itself is not pathological, and have questioned the need to medically classify and diagnose gender diversity [19, 20].

The International Classification of Disease (ICD) of the World Health Organisation (WHO) is used to code diagnoses and process payment for health care, especially in the private health care sector. It includes diagnoses for all body systems, whereas the DSM only categorises mental illness. In 1975, a diagnosis of “transsexualism” was introduced in the ICD-9 [14], and in the ICD-10, published in 1992, ﻿the diagnostic term was changed to “Gender Identity Disorder” [21]. In the ICD–11, ﻿this term will be changed to “Gender Incongruence” [22]. It will be relocated from the chapter on Mental and Behavioural Disorders to a new chapter, Conditions Related to Sexual Health. On 18 June 2018, the WHO published a version of ICD-11, with the press release stating “While evidence is now clear that it is not a mental disorder, and indeed classifying it in this way can cause enormous stigma for people who are transgender, there remain significant health care needs that can best be met if the condition is coded under the ICD” [23]. The ICD-11 was adopted at the World Health Assembly on 25 May 2019, for implementation in 2022 [24]. While such a diagnostic classification might be needed in order to access gender-affirming treatment, it is the view of many TGD activists and groups that it can further pathologize and stigmatise TGD identities [10, 25]. Although a strong argument has been made towards depathologisation, including in Southern Africa [19], some in the Southern African TGD community have also raised concerns regarding the depathologisation movement [26]. McLachlan [26] argues that “the African context may be more sympathetic towards a person who has a diagnosis and is identified as having a mental condition than a person who diverges from what is seen and/or constructed as the norm”. This remains a controversial topic with many different perspectives, ranging from no diagnostic category at the one end of the spectrum, to the middle ground of a diagnosis of “gender incongruence” in a separate chapter in the ICD-11, to retention as a mental health diagnosis as in the current DSM-V. ﻿Tensions continue to exist over how to classify “gender incongruence” to both depathologize gender diversity expressions and identities, while ensuring access to gender-affirming health care [15]. Regardless of if or how gender incongruence is classified within (or without) medical classification systems, TGD people have the right to receive health care that is affirming, respectful and non-judgmental, for which health professionals play a crucial role.

## Do TGD people experience gender-identity related health disparities?

Social determinants of health (SDOHs) are defined by the WHO as “the conditions in which people are born, grow, live, work and age” and that are “shaped by the distribution of money, power and resources.” [27]. Pega and Veale argue for the recognition of gender identity as a SDOH [28]. “Prejudice, stigma, transphobia, discrimination, and violence targeted at TGD people produce differential levels of social exclusion for populations defined by gender identity, including in health care settings. These social conditions disadvantage TGD people through social exclusion and privilege cisgender people through social inclusion, resulting in differential health outcomes. So, although gender identity in itself does not determine health, it socially stratifies the population into differential exposures to SDOHs such as transphobia”. This can be compared to other social stratifiers such as race or ethnicity, which are also considered SDOHs [28].

The health disparities are not inherent to TGD individuals but stem from structural factors such as government policy and hostile health care environments, as well as community and interpersonal factors such as social discrimination and rejection by families [12]. Such structural, community and interpersonal factors can contribute to a delay in accessing gender-affirming care [29, 30]. TGD people who belong to racial and ethnic minority groups face even more challenges [31]. Intersectionality acknowledges that identity is multidimensional and is impacted on by historical, structural, and cultural factors [32, 33]. Ng [33] eloquently explains that “Practicing medicine through the lens of intersectionality proactively considers patients’ diverse identities and how the sociocultural factors associated with membership in multiple minority groups can affect their health risks and health care experiences, and ultimately health decision making and health outcomes” [33]. It is thus important to keep in mind that despite a shared marginalised identity, TGD people are not a homogenous group, and that sub-groups and individuals may have different health care needs.

There are specific areas in which gender identity-related health disparities have been researched. In the section that follows, we will discuss mental health, Human Immunodeficiency Virus (HIV), violence and victimisation. This evidence on health disparities shows that there are specific gender identity-related issues that health professionals need to know about and that should be included in health science curricula.

## Mental health

A review of the health burden and needs of TGD populations globally reports that there is a significant mental health burden [12]. For example, estimates of depression prevalence were as high as 63% in a United States of America (USA) sample of 230 TGD women [34]. An Australian survey of 859 TGD young people found that 74.6% of participants had a diagnosis of depression and 72.2% an anxiety disorder. In this study, the incidence of self-harm was 79.7, and 48.1% of participants reported a suicide attempt in the past [35]. The authors point out that “the higher frequency of mental health difficulties than the general population is not because an individual identifies as TGD. Rather, these difficulties are largely caused by external factors – in other words, how the world perceives and treats transgender people” [35]. To make sense of the high rate of attempted suicides by TGD people, experiences of rejection and discrimination need to be considered as a key factor [36].

Meyer has described the concept of minority stress in LGB persons — explaining that “stigma, prejudice, and discrimination create a hostile and stressful social environment that causes mental health problems” [37]. Hendricks and Testa framed minority stress as a concept in TGD people [38], by applying the factors described by Meyer: “prior discrimination or victimization, expectations of future victimization or rejection, internalized transphobia, and resilience” [37, 38]. Firstly, the external events that impact on someone’s life as a result of their minority status such as discrimination and threats to their safety can negatively affect their mental health. The second factor is the anticipation and expectation that external stressful events will occur, leading to heightened vigilance. The negative expectations themselves can create distress for the person. The third factor is internalized transphobia, which can negatively affect someone’s ability to cope with external stressful events and ultimately reduces their resilience. This resonates with the description of TGD stigma by White Hughto, Reisner and Pachankis [39] as operating at structural, interpersonal and individual levels.

Importantly, Meyer [37] points out that not all of the effects of minority stress are negative, as members of minority groups can develop resilience. Hendricks and Testa [38] describe “group-level coping” in TGD persons, when they engage with other members of their minority group. Trans-specific social networks can create a supportive community that can buffer the effects of discrimination and violence. Riggs and Treharne (2017) add the theoretical framework of decompensation, described as “[ceasing]﻿ being able to compensate, [ceasing] being able to make up for the daily discrimination, [ceasing] being able to prop oneself up in the face of ideologies that render one’s existence unintelligible” [40]. This framework emphasises the need to challenge ideology and social norms that cause decompensation, as opposed to only focusing on individual resilience [40, 41]. Unfortunately, due to the lack of health professionals’ knowledge, and implicit or explicit prejudicial attitudes, the healthcare system often perpetuates the discrimination and marginalisation of TGD people within wider society, and this environment adds to, rather than alleviates, gender identity-related minority stress [42].

A study that compared the mental health of ﻿socially transitioned TGD children who are supported in their gender identity to that of cisgender children, found that depression rates were similar in both groups, and only slightly elevated anxiety rates were found amongst the TGD children [43]. Social transition can thus be regarded as a buffer against poor mental health. While there is a high prevalence of mental health challenges, there is evidence that gender-affirming hormone treatment can improve mental health [44–46].

## HIV

TGD women are disproportionately affected by HIV and other sexually transmitted infections [12]. A systematic review reported an odds ratio of 48.8 for HIV infection in TGD women compared with all adults of reproductive age across 15 countries [47]. A study of 230 TGD women in New York found that “gender abuse predicted depressive symptoms, and gender abuse combined with depressive symptoms predicted both high-risk sexual behaviour (unprotected receptive anal intercourse) and HIV” [34].

## Violence and victimisation

A high burden of violence and victimisation experiences in TGD people have been documented in research across the globe [12]. A WHO review reported that a high proportion of gender minority people experienced physical and sexual violence, motivated by bias or hate based on their gender identity [48]. This review found that “the prevalence of physical violence in TGD people ranged from 11.8% to 68.2% and sexual violence 7.0% to 49.1%”. A comparative study on being TGD in Europe that included 28 countries, analysed data from 6579 respondents [49]. While 54% of respondents stated that they had been discriminated against during the last year, 22% felt discriminated against in a health care setting [42]. A study of the effect of violence on TGD people, with a sample of 179 TGD women and 92 TGD men in Virginia [50] found that those who had experienced physical and/or sexual violence were significantly more likely to report a history of suicide attempts, alcohol abuse and illicit substance use. TGD individuals who present visibly as gender nonconforming have been shown to face even more discrimination compared to their gender conforming counterparts [51] and a UK study found that respondents currently undergoing a process of transition ﻿were significantly more likely to have reported experiencing physical and sexual harassment, compared to those who were proposing to undergo or had already undergone a process of transition [52]. In a survey of attitudes towards homosexuality and gender non-conformity in South Africa, 1% of respondents (*n* = 3079) agreed to the statement “I have physically hurt women who dressed and acted like men in public in the past year”, and between 6.2 and 7.4% of South Africans indicated that they might use violence against gender non-conforming people in the future [53]. Violence towards trans people is not only institutional and societal, but can be experienced within families, as described by Rogers [54] who found that family perceptions of shame and stigma can lead to transphobic ‘honour-based’ abuse.

## Do TGD people experience stigma and discrimination in health care settings?

TGD persons are more likely to face barriers when they try to access appropriate health care, compared to their cisgender peers [55]. There is evidence in the literature that transphobia in the health sector can lead to experiences of discrimination and stigma. Several USA studies of TGD persons reported negative health care experiences and found that knowledge gaps and discrimination contributed to a disparity in health care delivery [56–60]. A Canadian study of 923 TGD youth found that they described many past negative care encounters, with “uncomfortable and frustrating encounters with doctors” [61]. Two qualitative Swedish studies [62, 63] found that TGD persons experience estrangement in health care settings, due to lack of knowledge among health professionals. Participants described being treated as different, “to be regarded as a monkey in a cage appears to be very strenuous” [54]. In a UK study, 29 % of respondents (*n* = 411) felt that their gender identity was not validated as genuine in mental health settings and qualitative data indicated that some trans people felt that at gender identity clinics, the clinical sessions “ran counter to the preservation of their dignity and human rights” [64]. Negative experiences of gender diverse Australians were reported as physical healthcare being “invasive and sometimes abusive” [65]. There is limited research about TGD people published from the African continent and Asia. Qualitative studies in South Africa have reported that many of the TGD persons interviewed had experienced health workers as being discriminatory and hostile [66–68].

Negative health care experiences can be the result of subtle, apparently insignificant features of health care spaces and interpersonal interactions called microaggressions [69, 70]. Nadal et al. [70] define microaggressions as “subtle forms of discrimination, often unconscious or unintentional, that communicate hostile or derogatory messages, particularly to and about members of historically marginalized social groups” [70]. Although originally used to describe racial microaggressions [71], the theory was expanded to include other marginalised groups, including TGD people [70]. Health care spaces and providers often convey cisnormative microaggressions, which communicate to TGD people that “their identities, experiences, and relationships are abnormal, pathological, unexpected, unwelcome, or shameful” [69]. An example would be ﻿misgendering, a term meaning patients were misidentified or referred to by the incorrect pronoun [72].

## Gender and sexuality in health science education in relation to sexual and gender minority groups

Much of the negative attitudes of health professionals toward sexual and gender minority groups may originate from wider societal homophobia and transphobia. The paucity of education about LGBTQ health allows these notions to go unchallenged, thereby maintaining the heteronormative and cisnormative culture in health facilities [73]. In health sciences, the dominant pedagogical approach to sexuality has been biomedical. This emphasis leaves little space to interrogate the constructions of gender and sexuality through social dynamics [74]. Müller & Crawford-Browne [75] argue that “biomedical discourse bases its authority on empirical evidence – ‘objective’ scientific facts – and constructs people’s bodies as results of biological processes and determinations”. This biomedical approach makes it difficult to situate these bodies in their social context. Although more emphasis has been placed in recent years on the biopsychosocial approach, the health sciences have traditionally regarded bodies through a positivist lens that limits the extent to which socially constructed identities can be acknowledged [75].

It is essential for health sciences education to include critical reflection on the historical and contemporary hegemony of heteronormative and cisnormative discourses. This can assist both students and teachers to identify their discomfort with LGBTQ patients and reflect on how this could have originated in oppressive structures [76]. This can begin to address the root causes of the alienation experienced by TGD persons in health care settings, rather than just treating the symptoms.

## What are the gaps in curricula?

Several studies have been published internationally that describe the gaps in medical curricula. In a study of undergraduate medical education in the USA and Canada in 2009–2010, only 30.3% of the 150 medical schools surveyed reported teaching about gender transitioning [77]. Gaps in residency programs in the USA have been described for Emergency Medicine [78], Urology [79] and Plastic surgery [80]. A survey of 15 Australian and New Zealand medical schools found that teaching about gender and gender identity is varied across schools, with seven respondents (47%) unsure about what is taught [81]. In a United Kingdom study of medical students, participants were particularly unconfident on TGD health terminology and 72.9% felt “very unconfident” or “unconfident” deciding into which ward TGD patients should be admitted [82]. Canadian qualitative studies found a reported lack of knowledge regarding TGD health among family physicians [83] and mental health care providers [84]. A Canadian qualitative analysis of physician-side barriers to providing health care for TGD patients aptly titled “Completely out-at-sea with two-gender medicine”, found that a lack of knowledge made the clinical management of TGD patients more complicated [1]. In a survey of emergency medicine physicians in the USA, 82.5% reported that they did not receive formal training on TGD health care although 88% reported caring for this population [85]. A study of speech-language pathologists in four countries found that although TGD communication is within their scope of practice, 47% of respondents indicated that this was not included in their master’s curriculum [86]. A study of health professions education in South Africa and Malawi [87] found that there is little formal inclusion of LGBTQ health topics in nursing and medical curricula, and that educators who do teach LGB health topics reported doing so because “they felt personally compelled to include them”, not because this was supported or mandated institutionally. Topics related to TGD health and differences in sex characteristics were not covered by any of the participating educators [87].

An ethical discussion by Tomson [88] that compares the gatekeeping model and the informed-consent model of providing gender-affirming care provides an important perspective of how lack of knowledge of health professionals can lead to unethical care [88]. In the gatekeeping model, service providers make the assessment of whether or not a patient should be allowed access to gender-affirming care. Tomson [88] argues that this violates the principle of respect for autonomy. In contrast, the principle of autonomy is upheld by the informed-consent model. In this model, treatment is a cooperative effort between the patient and provider where well informed patients are the primary decision makers about their care [89]. A patient’s ability to make informed decisions about their health, e.g. starting hormone treatment, is enhanced by thorough education [89]. Furthermore, Tomson [88] argues that “since access to medical transition improves outcomes (particularly suicide risk) for TGD patients, limiting access to these interventions can be seen as harmful in and of itself, and as such, is a violation of the principle of non-maleficence”. When patients can decide on their own health care in an informed consent model, without factors such as race, social class or finance creating barriers to access, this promotes equity and fairness and upholds the principle of justice [88]. Although the informed consent model is used in some clinics [90], the gatekeeping model is still the mainstream treatment paradigm in many settings [91], which has implications for the role of health science education to promote an ethical model of care.

## What educational interventions have been described?

A recent scoping review of improving medical students’ and residents’ training and awareness of ﻿TGD health care found that consensus is lacking on exactly which educational interventions to use to address this topic [92]. Another review focusing on ﻿curricular initiatives that enhance student knowledge and perceptions of sexual and gender minority groups concluded that “multi-modal approaches that encouraged awareness of one’s lens and privilege in conjunction with facilitated communication seemed the most effective” [93]. The literature supports a shift toward longitudinally integrated and clinical skills based pedagogical interventions [92]. A 90 min workshop for psychiatry residents at Columbia university, USA, produced significant short-term increases in resident professionalism toward TGD patients [94]. However, on 90-day follow-up, this study did not find any statistically significant differences in perceived empathy, knowledge, comfort, and motivation for future learning, compared to baseline [94]. This highlights the limitations of one-time interventions and call for longitudinal programming to produce more durable improvements. Stroumsa et al. [95] caution that transphobia needs to be addressed specifically as a potential barrier to improved knowledge. Their study did not find any association between increased hours of education and improved knowledge, but found a negative association between transphobia and provider knowledge [95]. Gamble Blakey and Treharne [96] emphasize values cultivation as a starting point in educating about TGD healthcare, and argue that simply adding curricular content about gender-affirming care may not result in significant learning as this requires a sensitive and specific pedagogic discourse around values [97].

The Association of American Medical Colleges published an extensive resource for medical educators in 2014, titled “Implementing Curricular and Institutional Climate Changes to Improve Health Care for Individuals Who Are LGBT, Gender Nonconforming, or Born with DSD” [98]. It discusses the role of medical education and health care professionals in eliminating health disparities, lists professional competency objectives as well as discusses integrating competencies into medical school curricula [98]. This publication has been described by Donald et al. [29] as “representing a new frontier in medical education that attempts to redefine health to be inclusive of sexual orientation, gender identity, gender expression, and sex development—four intrinsic components of personhood” [29]. In the chapter on ﻿Trauma and Resilience, the authors emphasize that competence in providing care to diverse individuals requires more than an understanding of the causes of health disparities and to know to avoid microaggressions, making assumptions or discriminatory remarks: “It is imperative that health care providers learn how to promote resilience in the lives and families of individuals who are members of these groups so as to mitigate the effects of real and perceived trauma on risk behaviours and adverse health outcomes” [98].

There has been a recent proliferation of publications in professional journals to educate medical practitioners already in practice. These include the specialities of Endocrinology [99], Paediatrics [100–102]; Family Medicine [103, 104], Gynaecology [105], Psychiatry [106], Surgery [107, 108] and Anaesthesia [109]. Free e-learning courses have been developed such as “Primary Health Care for Trans, Gender Diverse & Non-binary People” [110] and “Caring for Gender Nonconforming young people” [111].

## Argument for including TGD health care in curricula

Winter argues that because “primary care is the most common point of contact that TGD people have with the health system, effective training for primary care providers through medical education and continuing professional development, is needed” [112]. Primary care providers can evaluate gender dysphoria and manage applicable hormone therapy [104]. In much of the world, specialist services for gender-affirming health care are not widely available, which reinforces the need for the training of primary care providers.

DasGupta and colleagues argue that incorporating social justice into the education of medical professionalism is critical [113]. A global consensus document on the social accountability of medical schools [114] includes statements that resonate with the need to include gender-affirming health in curricula, such as: “The medical school recognizes the various social determinants of health – and directs its education, research and service delivery programs accordingly,” and “the medical school recognizes the local community as a primary stakeholder and shares responsibility for a comprehensive set of health services to a defined population in a given geographical area, consistent with values of quality, equity, relevance”. A South African report, “Reconceptualising Health Professions Education in South Africa” [115] states that ﻿“the ultimate goal of health professions education is to produce knowledgeable, competent, relevant, socially accountable health care professionals capable of confidently and collaboratively promoting health and addressing the country’s burden of disease across the continuum of health care in the context of quality universal health coverage”. To be socially accountable, medical educators need to include the health needs of TGD people in medical curricula [29, 116]. The ethical imperative of the medical profession to reduce health care disparities and practice within the health care values of social justice, cultural humility and humanism has been highlighted by medical educators and researchers [98]. The World Medical Association (WMA) adopted a statement on TGD people in 2015 [117]. In this document, the WMA calls “for the provision of appropriate expert training for physicians at all stages of their career to enable them to recognise and avoid discriminatory practises, and to provide appropriate and sensitive transgender health care” [117].

## Conclusion

Whereas ideally gender should be viewed as a spectrum, and gender diversity as part of the diversity of humanity, in reality TGD persons often have very difficult lives due to not fitting into society’s cisnormative expectations [11, 12]. This leads to significant gender identity-related health disparities in the areas of mental health [34, 35], HIV risk [47], as well as violence and discrimination [48]. TGD people often experience stigma and discrimination in health care settings, which is a barrier to access to care [55]. Health professional attitudes and knowledge gaps contribute to and exacerbate these health disparities [56, 57]. The minority stress model describes how external stressors such as transphobic experiences can lead to anticipation of bad experiences, which can lead to avoidance of accessing health care [37, 38]. Several studies have described the gaps in undergraduate medical training [77, 81, 82] as well as residency training [78–80]. The gatekeeping model, where service providers decide who can access care, violates the ethical principle of respect for autonomy, while the informed-consent model upholds autonomy by empowering patients to make their own health care decisions [88]. As health science educators, representing a profession that has pathologized [10, 25], and continues to pathologize TGD identities [15], we have an ethical duty to include gender-affirming health in health science curricula [98, 116, 117] in order to prevent harm to TGD patients that our students will provide care for in the future.

## Data Availability

All data generated or analysed during this study are included in this published article

## Abbreviations

DSM: Diagnostic and Statistical Manual of Mental Disorders
HIV: Human Immunodeficiency Virus
ICD: International Classification of Disease
LGB: Lesbian, gay, bisexual
LGBT: Lesbian, gay, bisexual, transgender
LGBTQ: Lesbian, gay, bisexual, transgender, queer
SDOH: Social determinants of health
TGD: Trans and gender diverse
USA: United States of America
WHO: World Health Organisation
WMA: World Medical Association
